# Effect of the phenoxy groups on PDIB and its derivatives

**DOI:** 10.1038/srep35555

**Published:** 2016-10-19

**Authors:** Peng Song, Baijie Guan, Qiao Zhou, Meiyu Zhao, Jindou Huang, Fengcai Ma

**Affiliations:** 1 Department of Physics, Liaoning University, Shenyang 110000, China; 2 Liaoning Key Laboratory of Semiconductor Light Emitting and Photocatalytic Materials, Liaoning University, Shenyang 110036, PR China; 3 Institute of Theoretical Simulation Chemistry, Academy of Fundamental and Interdisciplinary Sciences, Harbin Institute of Technology, Harbin 150080, China; 4 School of Physics and Materials Engineering, Dalian National University, Dalian 116600, China

## Abstract

The anisotropic hole and electron mobilities in *N*,*N*′-3,4,9,10-perylenediimide-1,7-phenoxy (PDIB-2OPh) and *N*,*N*ʹ-3,4,9,10-perylenediimide (PDIB) were theoretically predicted using the Marcus–Hush theory. The substituent effect of phenoxy on their mobility rates, absorption spectra, electron affinities, and ionization potentials was explored. By comparing the simulated hole mobility in PDIB and PDIB-2OPh, it is found that the phenoxy rings act as spacers between adjacent stacking columns in the phenoxy-substituted derivatives. The increasement of the number of benzene oxygen groups leads to the absorption spectra red-shift of these molecular systems. This coincides with their change tendency of the adiabatic ionization potentials, vertical ionization potentials. However, the calculated adiabatic electron affinities and vertical electron affinities of *N*,*N*′-butyl-3,4,9,10-perylenediimide-1,6,7,12-phenoxy (PDIB-4OPh) are larger than those of PDIB;OPh. The steric effect in PDIB-4OPh is expected to cause space reversal and thus to changes in the properties of the molecule.

Organic semiconductor materials (OSCMs) have been widely used in organic optoelectronic devices such as organic field-effect transistors^1^,^2^, organic light-emitting diodes^3^,^4^,^5^,^6^ and organic photovoltaic cells^7^,^8^,^9^. Organic semiconductor materials possess a number of advantages over inorganic semiconductors, including flexibility, low weight, low cost, ease of processing, and versatility of chemical synthesis^10^. Exciton diffusion and charge transport are the most limiting factor for achieving higher device performance, and be at the heart of macroscopic operation of organic optoelectronic devices^11^,^12^,^13^. For further improving the efficiency of charge transport carrier mobility, more researchers have studied the anisotropic mobility of their materials^14^,^15^,^16^,^17^,^18^,^19^,^20^,^21^,^22^. It directly relates to the molecular organization. Clear anisotropic mobility mechanism for improving exciton diffusion and charge transport, provides the guideline in regarding molecular design and structure. The anisotropic mobility in rubrene single crystals was first explored by Sundar *et al*. in 2004^14^. The development of single-crystal, organic field-effect transistors has provided opportunity for exploring the intrinsic properties of organic materials, namely, angular resolution mobility anisotropy^23^,^24^ and the Hall effect^25^.

Very recently, three perylenediimide derivatives with different structural motifs, named *N*,*N*′-butyl-3,4,9,10-perylenediimide (PDIB), bay-area-substituted *N*,*N*′-butyl-3,4,9,10-perylenediimide-1,7-phenoxy (PDIB-2OPh), and bay-area-substituted *N*,*N*′-butyl-3,4,9,10-perylenediimide-1,6,7,12-phenoxy (PDIB-4OPh), were synthesized. The effect of molecular stacking on favorable exciton diffusion of these organic semiconducting materials was experimentally studied using single-crystal charge-transfer interfaces, which fabricated through distinct charge percolation networks. Photoconductivity measurements show that high-quality donor–acceptor interfaces yield a wide spectral response in the visible range. Most importantly, photoresponse measurements reveal that this interfaces present higher photocurrent^26^. These results clearly indicate the stacking contact along different directions can significantly increase exciton diffusion and electronic transport is not compromised. However, the charge transport, especially the anisotropic hole and electron mobility through distinct stacking connection, in these organic semiconducting materials is still unclear. Assisted to exciton diffusion, it is essential for further improving the efficiency of OSCMs.

In theoretically aspect, two main widely used models for describing charge transport in OSCMs are band-like model and the hopping model, respectively^27^. Usually, the band-like model is valid in very low temperature. In room temperature, charge transport between neighboring molecules is thermally favored and the sequential polaron hopping model is reasonable. The hopping rate can be well predicted using Marcus-Hush theory^28^. It directly relates to the intermolecular electronic coupling and internal reorganization energy^29^. By employing the simulation methods, Wen *et al*. investigated the anisotropic hole and electron mobility of n-type electron-withdrawing substituted perylene bisimide (PBI) derivatives. It is found that the well controlled direction of crystals is benefit for improving the material performance^27^. Also they used rigorous quantitative functions to characterize electronic coupling oscillation of slipped-cofacial stacking acene derivatives. The above similar strategy is adopted to establish the structure-activity related charge transport motion^29^.

Inspired by the above analysis, in the present study, we used Marcus–Hush theory based on the quantum chemical method to obtain various parameters for simulating the anisotropic hole and electron mobility of three perylenediimide derivatives (molecular structures are shown in Fig. 1). In particular, this theoretical model was used to quantitatively express the angular resolution anisotropic mobilities as a mobility orientation function *μ*_Φ_(*V*, *λ*, *r*, *θ*, *γ*, *Φ*). We then observed the molecular-packing architecture and calculated the underlying electronic properties, namely, electronic coupling (*V*) and reorganization energies (*λ*). Finally, we examined the effect of phenoxy on the mobility rate, absorption spectrum, electron affinity, and ionization potential of the molecules.

## Marcus–Hush Theory

First-principles quantum mechanics (QM) combined with Marcus–Hush theory was applied to simulate the molecules. For an organic crystal to which the hopping mechanism at room temperature is applied, the nonadiabatic electronic hopping rate (*W*) is given by the Marcus–Hush equation^28^,^30^:


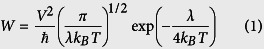


where *V* is the electronic coupling between neighboring molecules in the organic single-crystal, *T* is the temperature, and *k*_B_ is the Boltzmann constant.

Under the assumption of no correlation between hopping events, *W* between neighboring molecules in the organic single-crystal leads to the diffusion coefficient, *D*:


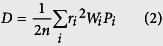


where *n* is the spatial dimensionality, and *i* represents a specific hopping pathway with hopping distance *r*_*i*_ (the intermolecular center-to-center distance of different dimer types). *P* is the hopping probability, which is calculated as follows:


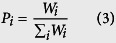


Evaluation of the diffusive mobility due to charge hopping *μ* using the Einstein relation gives the bulk (isotropic) mobility of the material (*μ*):


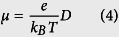


The magnitude of the field-effect mobility in a transistor channel depends on the specific surface of the organic crystal. The mobility of components for each surface may be analyzed in terms of angles (*γ*_i_) between the charge hopping pathways and the plane of interest (*W*_*i*_*r*_*i*_ cos *γ*_*i*_)^31^. In general, unsubstituted *π*-conjugated molecules crystallize into a layered herringbone packing, which allows two-dimensional transport within the basal stacked organic layers; transport between layers, however, is less efficient. *γ*_*i*_ for the hopping paths in the basal stacked layers is 0°.

**Figure 1:**
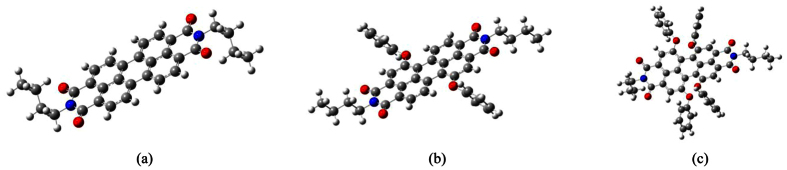
Molecular structures of PDIB (**a**), PDIB-2OPh (**b**), and PDIB-4OPh (**c**).

**Figure 2:**
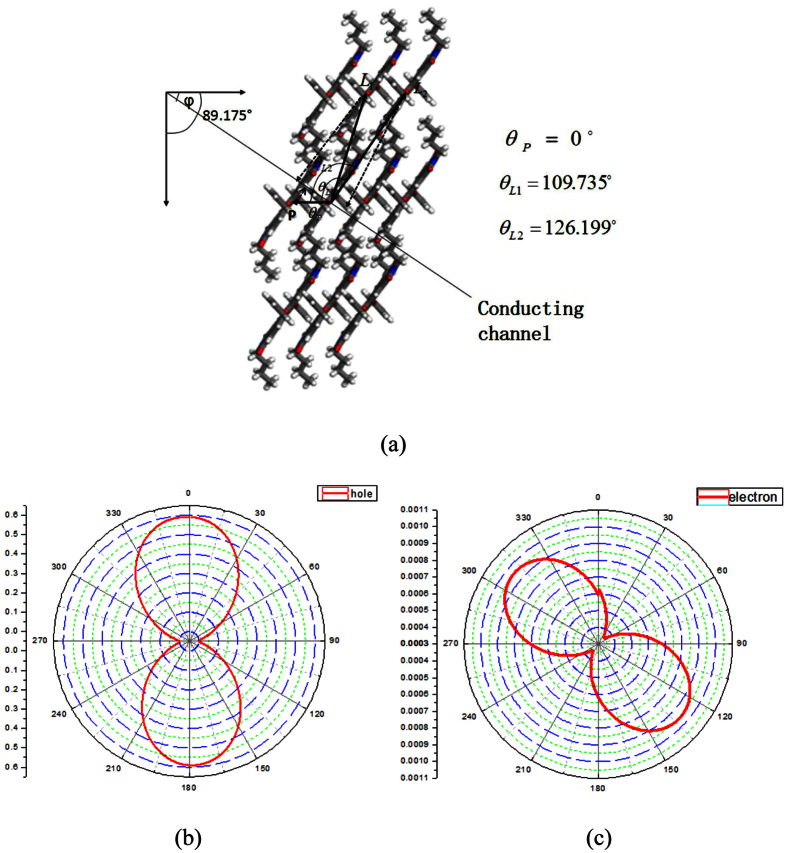
(**a**) Different hopping paths projected to a transistor channel in the *a*–*b* plane of single crystals of PDIB-2OPh. *θ*_*L*1_, *θ*_*L*2_, and *θ*_*P*_ are the angles of the *L*_1_, *L*_2_, and *P* dimers relative to the reference crystallographic *a* axis; *φ* is the angle of a conduction channel relative to the reference crystallographic *a* axis. (**b**,**c**) The angular resolution mobilities for hole and electron transport along the *a*–*b* plane in PDIB-2OPh.

**Figure 3:**
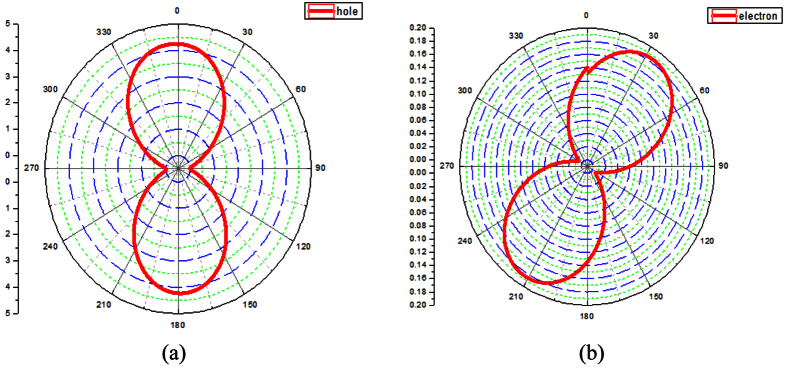
(**a**,**b**) Angular resolution mobilities for hole and electron transport along the *a*–*b* plane of PDIB.

With the basal plane as the reference, *Φ* is the orientation angle of the transistor channel relative to the reference axis (such as the crystallographic axis *a*, *b*, or *c*), and {*θ*_*i*_} are the angles of the projected hopping paths of different dimer types relative to the reference axis. Thus, the angles between the hopping paths and the conducting channel may be expressed as *θ*_*i*_ − *Φ* (Figs 2a and 3a). We can then project the hopping paths onto the different transistor channels (*W*_*i*_*r*_*i*_ cos *γ*_*i*_ cos(*θ*_*i*_ − *Φ*)). In the typical layered herringbone packing, neighboring molecules in the same layers can be transverse dimers (T) and parallel dimers (P), as illustrated in Fig. 1. For ideal high-purity crystals, the orientations of the surrounding molecules are identical; in this case, Eqs (2)–(4), 3, 4 lead to the orientation function describing the mobility in a specific conducting direction on a specific surface in the organic crystal^32^:


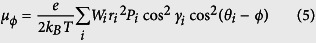


where *P*_*i*_ cos^2^ *γ*_*i*_ cos^2^(*θ*_*i*_−*Φ*) describes the relative hopping probability of various dimer types in the specific transistor channel; *r*_*i*_, *γ*_*i*_, and *θ*_*i*_ are determined by the molecular packing architecture in the organic crystal; other terms are defined as above. In Eq. (5), the mobility in a unique conducting direction is determined by all related hopping pathways. It is a combined effect of *V* from different hopping pathways in the organic material. As a specific *Φ* corresponds to a specific conducting direction, Eq. (5) is a one-dimensional model. Thus, *n* in Eq. (2) is taken to be 1 in the derivation of Eq. (5).

Equation (5) provides an analytical function for determining the angular-resolved anisotropic mobilities for any type of organic semiconductor by relating the crystal packing and *V* to *Φ*. We describe the mobility as a function of the orientation angle of the transistor channel in a plane, assuming that *μ′*(*Φ*) = 0. The conditions *μ′′*(*Φ*) > 0 and *μ′′*(*Φ*) < 0 respectively define directions for the conducting channels with the highest and the lowest mobility in the plane. When *μ′*(*Φ*) = 0,


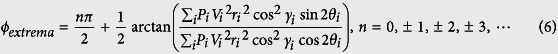


When the orientation angle of the transistor channel equals the *Φ* extrema, the highest and lowest mobilities in the plane may be calculated by substitution of the *Φ* extrema values into Eq. (5).

The parameters *V* and *λ* determine the relations in Eqs (1)–(6), 2, 3, 4, 5, 6, both of which can be derived from first-principles calculations. We use the adiabatic potential energy surfaces method to calculate *λ*^8^. The geometries of the isolated molecules in the neutral and in the cationic or anionic states were optimized by using DFT with the B3LYP functional and with the 6-31G^∗∗^ basis set. For a neutral monomer A, the reorganization energies for hole transport and electron transport are as follows:


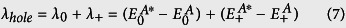



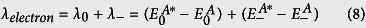


where 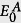 and 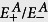 denote the energies of the neutral monomer and of the cationic or anionic monomers in their respective optimized geometries; 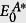 and 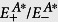 respectively denote the energies of the neutral monomer and of the cationic or anionic monomers with the corresponding geometries. Calculations for electronic coupling of the dimers, whose geometries are selected from the experimental X-ray crystal structures^26^, were performed by using the PW91/TZ2P flavors of density functional theory (DFT) method^32^,^33^, as implemented in the ADF program^34^. 4V may be calculated from the spatial overlap (*S*_*RP*_), charge-transfer integral (*J*_*RP*_), and site energies (*H*_*RR*_, *H*_*PP*_):


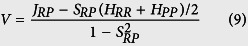


Assuming that *h*_ks_ is the Kohn–Sham Hamiltonian for the dimer system, which consists of two monomers and that 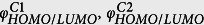 are the highest occupied molecular orbitals or the lowest unoccupied molecular orbitals of the two monomers, we can obtain *S*_*RP*_, *J*_*RP*_, *H*_*RR*_ and *H*_*PP*_ of p-type organic materials for the calculation of *V*:


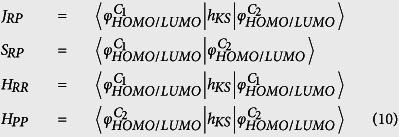


## Results and Discussion

### Effect of phenoxy-substituted PDIB based on the mobility rate

The calculated mobilities though random walking traces of charge carriers, were performed by using a statistical method and cannot reflect the anisotropic property of charge transport. For the PDIB-2OPh crystal, the *a*–*b* plane was chosen as the surface for anisotropic charge transfer. The crystallographic *b* axis was set as the reference axis, as shown in Fig. 2a. Two kinds of hopping routes (*L* and *P*) were arranged in the *a*–*b* plane. The hopping paths *L*_1_, *L*_2_, and *P* are clearly on the *a*–*b* plane, and *γ*_*i*_ is 0 for all of them. The orientation angle of the conduction channel relative to the reference *b* axis is *φ*. The angles between the hopping routes *L*_1_, *L*_2_, and *P* and the reference *a* axis are *θ*_L1_, *θ*_L2_, and *θ*_*P*_, respectively, with values of 109.735°, 126.199°, and 0°, respectively. The angles between the hopping routes *L*_1_, *L*_2_, and *P* and the conducting channels are *θ*_L1_−*φ*, *θ*_L2_−*φ* and *θ*_P_−*φ*, respectively. Given the molecular packing parameters (r, *θ*, and *γ*) and the charge-transfer parameters (*V* and λ), we can obtain an orientation mobility function *μ*_(*φ*)_ for holes and electrons along the *a*–*b* plane using Eq. (5). Charge-transfer parameters (*V* and *λ*) and one-dimensional drift mobilities for hole and electron transport along different transfer routes are listed in Table 1.

*μ*_(*φ*)_ for hole and electron transport along the *a*–*b* plane is expressed as Eqs (11) and (12), respectively.


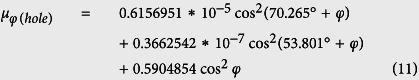



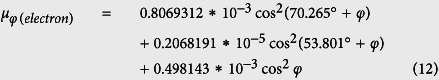


The maximum hole mobility is achieved when *φ* = 0° and 180° (Fig. 2b). The maximal mobility for hole transport along the *a*–*b* plane is 0.59 *cm*^2^*V*^−1^*s*^−1^. The maximum electron mobility is achieved when *φ* = 128° and 308° (Fig. 2c). The maximal mobility for electron transport along the *a*–*b* plane is 0.95 × 10^−3^ *cm*^2^*V*^−1^*s*^−1^.

In general, the hopping pathway with the largest electronic coupling is the direction of the highest mobility. Indeed, the highest mobility direction for holes is along P dimers with the largest electronic coupling. However, the predicted direction for the highest mobility of electrons is not along the P dimer with the largest electronic coupling. We can explain this phenomenon from the mobility orientation function. The electronic coupling of P dimers for hole mobility are much larger than that of other dimers. In contrast, the electronic couplings of *P*, *L*_1_, and *L*_2_ dimers for electron mobility are close. Because of the different parameters for holes and electrons, the mobility orientation function directly leads to a difference between the mobility anisotropic curves for holes and electrons (Fig. 2b,c).

The calculated mobilities of holes and electrons in the *a*–*b* plane of the PDIB molecule are shown in Fig. 3. when *φ* = 0° and 180°, the hole mobility reaches the maximum value (Fig. 3); the maximum mobility along the *a*–*b* plane is 4.23 cm^2^V^−1^s^−1^. At *φ* = 35° and 215°, the maximum electron mobility along the *a*–*b* plane is 0.17 cm^2^V^−1^s^−1^.

From the mobility chart, we can find that the hole mobilities along the *a*–*b* plane of PDIB-2OPh and PDIB crystals are 0.59 and 4.23 cm^2^V^−1^s^−1^, respectively. These values suggest that phenoxy rings act as spacers between adjacent stacking columns in the phenoxy-substituted derivatives. In the present study, we confirm this hypothesis from the optical properties.

### Effect of phenoxy-substituted PDIB based on absorption spectra

**Figure 4:**
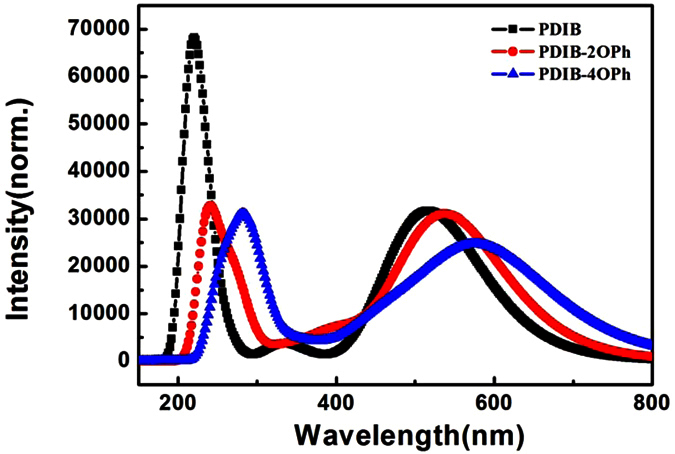
Absorption spectra of PDIB and its derivatives.

On the basis of the vertical transition mechanism of electron absorption, the electronic absorption spectra of PDIB, PDIB-2OPh, and PDIB-4OPh were simulated by employing the time-dependent DFT at B3LYP/6-311G level. These calculations are carried out with Gaussian 09 program package^35^. As can be seen from Fig. 4, these three perylenediimide derivatives present broad and strong absorption in the UV-Visible region, which can be assigned to π→π^∗^ transition due to the center π-conjugated perylenediimide core for these compounds. Attaching more electron-donating benzene oxygen groups on the perylenediimide skeleton leads to the red-shift of absorption spectra. This is in good agreement with the corresponding experimental results^27^. The main absorption bands are peaked at 223.23 and 516.71 nm, 239.27 and 538.34 nm, 275.45 and 586.34 nm, for PDIB, PDIB-2OPh and PDIB-4OPh, respectively. With the increase in number of phenoxy rings, the π-conjugation properties of molecular systems enhance, which results in the energy decrease needed for optical transition. It indicates that the organic π-conjugated PDIB and its derivatives, as the OSCMs with better photoconductivity and photoresponse characters, have potential application as the photoactive electron donor in organic solar cell, in term of their wide spectral response in the visible range.

### Effect of phenoxy-substituted PDIB based on its ionization potential and electron affinity

The adiabatic ionization potentials (AIPs), vertical ionization potentials (VIPs), adiabatic electron affinities (AEAs), and vertical electron affinities (VEAs) of three perylenediimide derivatives are calculated with DFT method at the level of B3LYP/6-311G, as shown in Table 2.

**Figure 5:**
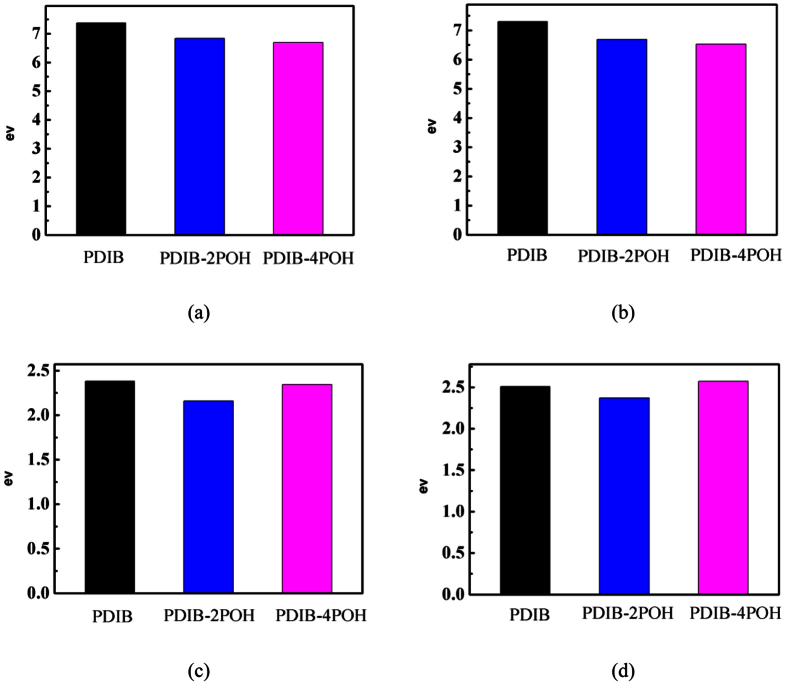
Values of (**a**) VIP, (**b**) AIP, (**c**) VEA, and (**d**) AEA of PDIB and its derivatives.

In order to facilitate observation, a longitudinal comparison of the VEAs, AIPs, AEAs, and VIPs of the three compounds were performed and presented in Fig. 5. The electron affinity and ionization potential directly relate to the overcoming energy barrier ability of electrons and holes injecting the electrodes or virtual orbitals. By substituting electron-donating benzene oxygen groups, VIP and AIP similarly decrease with the increase in number of phenoxy groups in the molecular systems. However, the AEA and VEA for PDIB-4OPh show the opposite trend to VIP and AIP results. It is reasonably believed that this trend is due to addition of four phenoxy groups to the PDIB molecule. The steric effect in PDIB-4OPh is expected to cause space reversal and thus to changes in the properties of the molecule. In general, the substituent is disadvantageous for p-type semiconductors in respect of the ionization potential. The decrease of ionization potential impedes the holes injecting into empty orbitals of semiconductors. This indicates that PDIB is more potential as a p-type organic semiconductor than the phenoxy-substituted derivatives.

## Conclusion

A simulation model based on first-principles QM calculations and the Marcus–Hush theory was used to study the charge transport along the hopping paths and to predict the anisotropic mobilities in PDIB and PDIB-2OPh. We then determined the molecular packing parameters *r*, *θ* and *γ*, as well as the electronic properties *V* and *λ*. Because the different dimers of the organic crystals are similar in terms of electronic coupling, the hopping pathway with the largest electronic coupling may not be the direction of the highest mobility. We determined the effect of the phenoxy group on PDIB and its derivatives by studying the molecules. In particular, we found that the hole mobility in PDIB and PDIB-2OPh molecules is higher than the electron mobility. The maximum hole mobility in PDIB is 94 times that of the electron maximum mobility, showing that the two molecules are suitable p-type organic semiconductors. We compared the hole mobilities in PDIB and PDIB-2OPh and thus found that phenoxy rings act as spacers between adjacent stacking columns in the phenoxy-substituted derivatives. We confirmed this conclusion from their optical properties. By comparing the absorption spectra of PDIB, PDIB-2OPh, and PDIB-4OPh, we showed that an increase in the number of phenoxy groups in the molecular system decreases the maximum spectral absorption and causes a spectral red-shift. Comparison of VIPs, AIPs, VEAs, and AEAs of the three molecules revealed that IPs decrease with the increase in the number of phenoxy groups. EAs for PDIB-4OPh, however, increases. We believe that this trend is due to the changing of the configuration by adding the phenoxy to the PDIB molecule. Our simulated results indicate that PDIB is more potential as a p-type organic semiconductor than the phenoxy-substituted derivatives.

## Additional Information

**How to cite this article**: Song, P. *et al*. Effect of the phenoxy groups on PDIB and its derivatives. *Sci. Rep.* **6**, 35555; doi: 10.1038/srep35555 (2016).

## Figures and Tables

**Table 1:** Calculated transfer integrals and reorganization energies for hole and electron transport, as well as one-dimensional drift mobilities along different transfer routes in the *a*–*b* planes.

Crystal plane	Transfer routes	Transfer distance *r*_*i*_ (Å)	Transfer integral *V*_*i*_ (eV)		Drift mobility *μ*_*i*_ (cm^2^V^−1^s^−1^)	
			Hole	Electron	Hole	Electron
*a*–*b* plane	P	4.746	0.10393	0.01041	0.5904854	0.000498143
	L1	13.513	0.0035	0.00696	6.15695 × 10^−6^	0.000806931
	L2	15.762	0.0009	0.00145	3.66254 × 10^−8^	2.06819 × 10^−6^
*λ* (eV)	0.26477217 (holes)		0.45351574 (electrons)			

**Table 2:** Values of AIP, VIP, AEA, and VEA of PDIB and its derivatives.

Molecular crystal	VIP (eV)	AIP (eV)	VEA (eV)	AEA (eV)
PDIB	7.3721	7.2991	2.3807	2.5082
PDIB-2OPh	6.8348	6.6916	2.1593	2.3719
PDIB-4OPh	6.6895	6.5322	2.3444	2.5708
